# Emotional State and Salivary Inflammatory Markers in Endometriosis Associated Pelvic Pain: A Pilot Study Comparing Chronic and Cyclic Patterns

**DOI:** 10.3390/cimb48080817

**Published:** 2026-08-12

**Authors:** Mario de Jesús Meingüer-Cuevas, Miroslava Avila-García, Aurora Espejel-Núñez, Arturo Flores-Pliego, Ignacio Camacho-Arroyo, Héctor Romo-Parra, Tahiri Mendoza-Hernández, Oliver Cruz-Orozco, Brenda Sánchez-Ramírez, Roberto Silvestri-Tomassoni, Omar Villa-Robledo, Javier Mancilla-Ramírez, María del Pilar Meza-Rodríguez

**Affiliations:** 1 Sección de Posgrado e Investigación, Escuela Superior de Medicina, Instituto Politécnico Nacional, Mexico City 11340, Mexico; psic.mmc@hotmail.com (M.d.J.M.-C.); mavilagarcia@gmail.com (M.A.-G.); tahiri.mendoza.05@gmail.com (T.M.-H.); 2 Departamento de Investigación en Psicología, Instituto Nacional de Perinatología, Montes Urales 800, Mexico City 11000, Mexico; 3 Departamento de Infectología e Inmunología, Instituto Nacional de Perinatología, Montes Urales 800, Mexico City 11000, Mexico; 4 Departamento de Inmunobioquímica, Instituto Nacional de Perinatología, Montes Urales 800, Mexico City 11000, Mexico; auro.espejel@gmail.com (A.E.-N.); arturofpliego@gmail.com (A.F.-P.); 5 Unidad de Investigación en Reproducción Humana, Instituto Nacional de Perinatología-Facultad de Química, Universidad Nacional Autónoma de México, Mexico City 04510, Mexico; camachoarroyo@gmail.com; 6 Departamento de Neurofisiología, Instituto Nacional de Neurología y Neurocirugía, Av. Insurgentes Sur 3877, Mexico City 14269, Mexico; hector.romo.parra@gmail.com (H.R.-P.); villa.omar@uabc.edu.mx (O.V.-R.); 7 Departamento de Ginecología Quirúrgica, Instituto Nacional de Perinatología, Montes Urales 800, Mexico City 11000, Mexico; oliverpco@gmail.com (O.C.-O.); dra.bsr@gmail.com (B.S.-R.); drsilvestri@hotmail.com (R.S.-T.); 8 Centro Cívico, Facultad de Medicina y Nutrición, Universidad Autónoma de Baja California, Mexicali 21000, Mexico; 9 Hospital de la Mujer, Secretaría de Salud, Salvador Díaz Mirón 374, Miguel Hidalgo, Mexico City 11340, Mexico

**Keywords:** endometriosis, anxiety, depression, women’s health, inflammation, cytokines

## Abstract

Women with endometriosis experience chronic cyclic pelvic pain (CCPP) or chronic persistent pelvic pain (CPPP), both of which may be incapacitating even after treatment. Emotional dysregulation in endometriosis impedes patient recovery. This study evaluated the relationships among emotional state, pain perception, and inflammatory biomarkers (IL-1β, IL-6, and TNF-α) in women with endometriosis presenting with CCPP or CPPP. An exploratory, observational, descriptive, cross-sectional, comparative with repeated sampling study was conducted with 52 women diagnosed with endometriosis and experiencing either CPPP or CCPP. Participants completed a psychometric battery including the State-Trait Anxiety Inventory (STAI), Beck Depression Inventory (BDI-II), Goldberg General Health Questionnaire (GHQ-30), Hospital Anxiety and Depression Scale (HADS), and Mini-Mental State Examination (MMSE). Pain perception was assessed using the Wong–Baker Pain Rating Scale (FACES). Saliva samples were collected at baseline, during stressor and recovery phases, and concentrations of IL-1β, IL-6, and TNF-α were determined by ELISA. Fifty-two women with endometriosis were included (CPPP: *n* = 33; CCPP: *n* = 19). No significant between group differences were observed in emotional state (HADS: *p* = 0.682; BDI: *p* = 0.842), anxiety (STAI-State: *p* = 0.086; STAI-Trait: *p* = 0.615), general distress (GHQ-30: *p* = 0.730), or pain intensity (FACES: *p* = 0.705). The prevalence of depressive symptoms did not differ between groups (CPPP: 69.7% vs. CCPP: 73.7%; χ^2^ = 0.093, *p* = 0.760). Salivary cytokine levels (IL-1β, IL-6, TNF-α) were comparable between groups across all measurement conditions. Spearman correlations revealed uncorrected significance between TNF-α and emotional distress: basal TNF-α correlated inversely with HADS (ρ = −0.292, *p* = 0.031) and BDI (ρ = −0.278, *p* = 0.041); TNF-α under stress correlated with HADS (ρ = −0.329, *p* = 0.014) and GHQ-30 (ρ = −0.271, *p* = 0.046); and TNF-α during recovery correlated with GHQ-30 (ρ = −0.363, *p* = 0.006). These findings indicate that CPPP and CCPP were not associated with statistically significant differences in emotional states or salivary cytokine profiles at the group level. Exploratory pos hoc analyses suggested that pain pattern may moderate the association between TNF-α and psychological burden, particularly in the CCPP subgroup; however, these findings require confirmation in larger studies with prespecified analyses. Exploratory analyses suggested patterns between salivary inflammation and psychological burden; however, these findings should be interpreted cautiously because of the small subgroup size, multiple comparisons, and lack of a matched healthy control group.

## 1. Introduction

Endometriosis is a gynecological disease associated with infertility of unknown origin. It affects between 10 and 15% of women of reproductive age worldwide [1]. In Mexico, it is estimated to affect more than seven million women, many of whom are misdiagnosed because they consider menstrual pain to be normal [2]. The main symptoms are chronic and persistent pelvic pain, which, in some cases, can be disabling even after surgery and drug treatment. Emotional dysregulation triggered by chronic pain can hinder patient recovery [3]. To address this, interdisciplinary treatments incorporating physical and psychological rehabilitation techniques have been implemented to alleviate pain in patients with endometriosis; however, few studies have analyzed the effect of psychological interventions on pain and related biochemical markers in patients with endometriosis [4,5].

Endometriosis is a medical condition in which glands and stroma are present outside the uterine cavity. These glands and stroma can be found in regions such as the bladder, ureters, fallopian tubes, peritoneum, ovaries, and even extra-pelvic sites [6]. This condition affects 50% of infertile women worldwide, making it the most common gynecological disease in women of reproductive age and perimenopausal women [7]. The two main clinical and physiological characteristics that accompany a diagnosis of endometriosis are chronic pelvic pain that can be permanent or cyclical and severe enough to cause incapacitation [8]; this endometriosis symptoms can have a devastating impact on patients’ functional status, lifestyle, social support networks, and relationships with partners and family [9].

In Mexico, determining the prevalence of endometriosis has been challenging because many patients do not undergo imaging studies such as laparoscopy. These studies could aid in diagnosis and enable timely treatment for managing the disease. However, epidemiological reviews conducted at the Instituto Nacional de Perinatología (INPer) estimate the incidence of endometriosis to be 34.5% among women diagnosed with primary or secondary infertility [10].

The pain associated with endometriosis is usually quite intense and often turns off [11]. It can occur cyclically, before, during, and after menstruation (known as dysmenorrhea), or during sexual intercourse (known as dyspareunia). Chronic pelvic pain (CPP) may also be present [12,13,14]; CPP is defined as constant and recurring discomfort in the pelvic area for at least six months not associated with malignancy, as is the case with endometriosis [15]. Central Sensibilization can be explained by these pain symptoms like chronic pelvic pain, dysmenorrhea, dysuria and dyschezia; in particular, it appears more common in patients with moderate-to-severe chronic pelvic pain [16]. From medical and psychological perspectives, CPP is the main clinical problem of endometriosis. It affects women’s quality of life, making it difficult to perform work and/or school activities. CPP also interferes with women’s social life and development of interpersonal relationships [12,17]. This pain particularly affects couples’ lives, as it can lead to the avoidance of or interruption during sexual encounters due to CPP [18,19].

Furthermore, the treatments used for endometriosis can cause significant absenteeism from work, which can lead to loss of employment and income. These treatments also reduce the possibility of attending social events, increasing feelings of frustration and social isolation [20]. Consequently, patients with endometriosis often experience high levels of depression and anxiety, which are commonly associated with uncertainty about the disease’s progression and the future of their lives [21,22].

Given the significance of CPP in women diagnosed with endometriosis, the International Association for the Study of Pain [23] emphasizes the necessity of an interdisciplinary approach to managing this condition that considers its biopsychosocial nature [24]. According to this model, pain is a subjective experience rooted in a neurobiological system that enables us to perceive, distinguish, evaluate, and feel a nociceptive event [25]. In this model, social support or isolation [21,25] and psychological states (thoughts, emotions, and behaviors) can exacerbate or diminish the nociceptive signal through brain regions such as the anterior insula and anterior cingulate cortex [25,26].

Due to the biopsychosocial nature of pain, using biomarkers to study this phenomenon is becoming more common. These biomarkers examine how the nervous, endocrine, and immune systems regulate the nociceptive experience [27]. To characterize pain biomarkers, researchers have studied different proinflammatory cytokines, such as interleukin-1β (IL-1β), interleukin-6 (IL-6), tumor necrosis factor-α (TNF-α), and C-reactive protein, due to their involvement in the chronicity of pain and their effects on anxious and depressive symptoms through alterations in the dopaminergic and serotonergic systems [28,29]. Additionally, elevated levels of circulating and local cyclooxygenase-2, TNF-α, prostaglandin-E2, growth factors, cytokines, and angiogenic factors are implicated in proinflammatory signaling [30]. Increased IL-1α, IL-6, and IL-8 promote cell growth and angiogenesis, and fibronectin allows attachment of ectopic cells [31]. Genetic and epigenetic changes have been studied in the pathogenesis of endometriosis [32], which means that 50% of cases of endometriosis lesions are linked to hereditary factors [33].

Modulation of the pain response includes an increase in cortisol levels and a decrease in sulfated dehydroepiandrosterone (DHEA-S) levels, which intensifies and maintains the nociceptive experience [34,35].

However, prolonged release of pain biomarkers can compromise the functioning of the autonomic nervous system, and elevated levels of circulating cortisol and catecholamines generate an increase in proinflammatory cytokines [36]. Therefore, evaluating soluble mediators involved in chronic pain alterations is key to developing practical, comprehensive care proposals for patients with endometriosis and CPP [37].

According to the International Association for the Study of Pain, proper management of patients with chronic pain requires psychological intervention as a key element of recovery [38,39,40,41].

A systematic review found that, on average, scores on pain assessments using the visual analog scale (VAS) decreased from four to three points. However, this reduction was not significant in all cases. This underscores the necessity of more effectively assessing the pain construct by considering its characteristics (location, duration, and sensory and affective description), the patient’s status, and their ability to respond to pain. It is crucial to consider the functional impact on daily activities and sleep hours, not just the intensity of the nociceptive stimulus [42,43].

## 2. Materials and Methods

### 2.1. Ethical Review and Sample Collection

**Figure 1:**
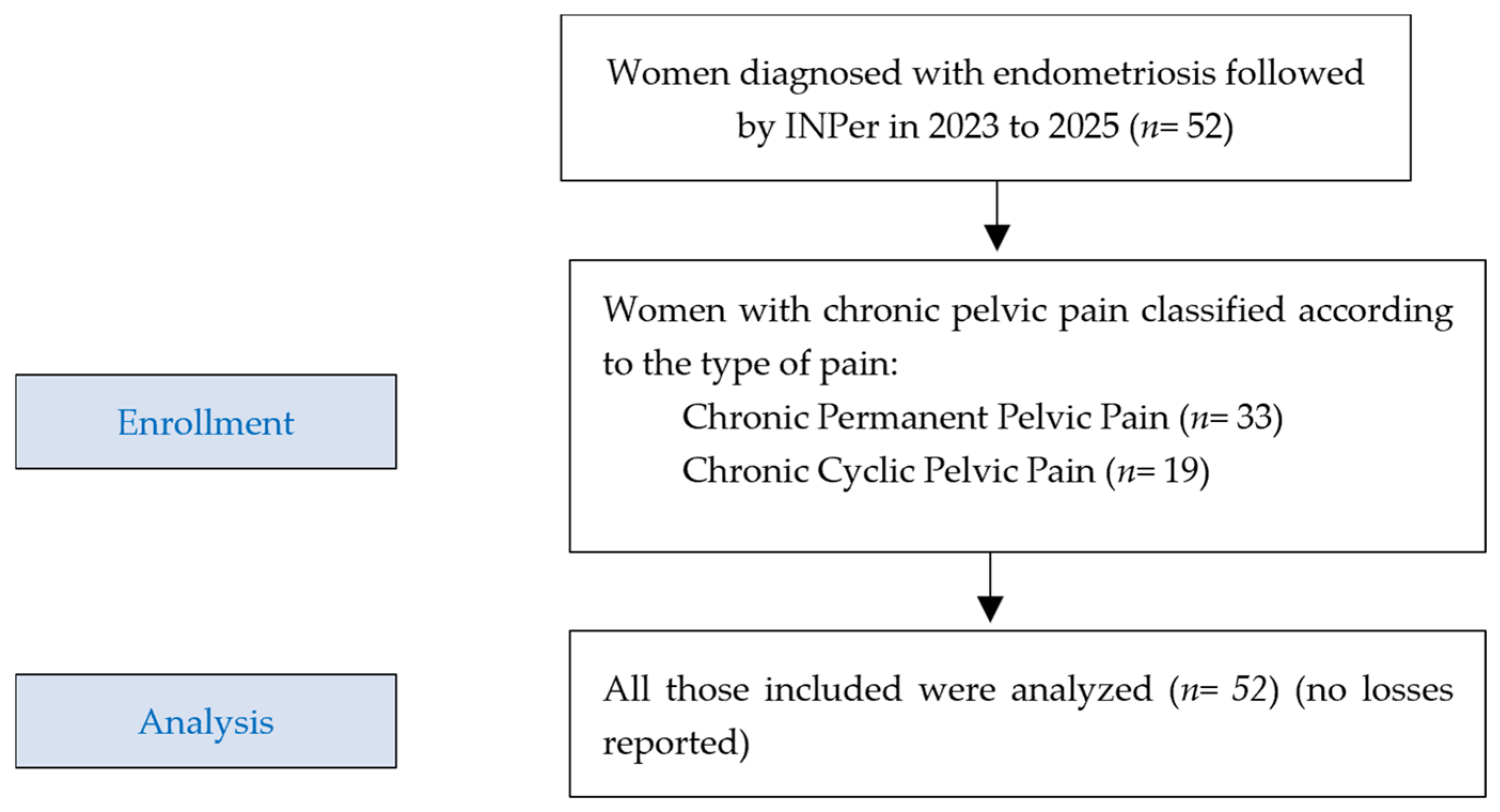
STROBE-style flow chart describing recruitment of subjects in the study.

**Figure 2:**
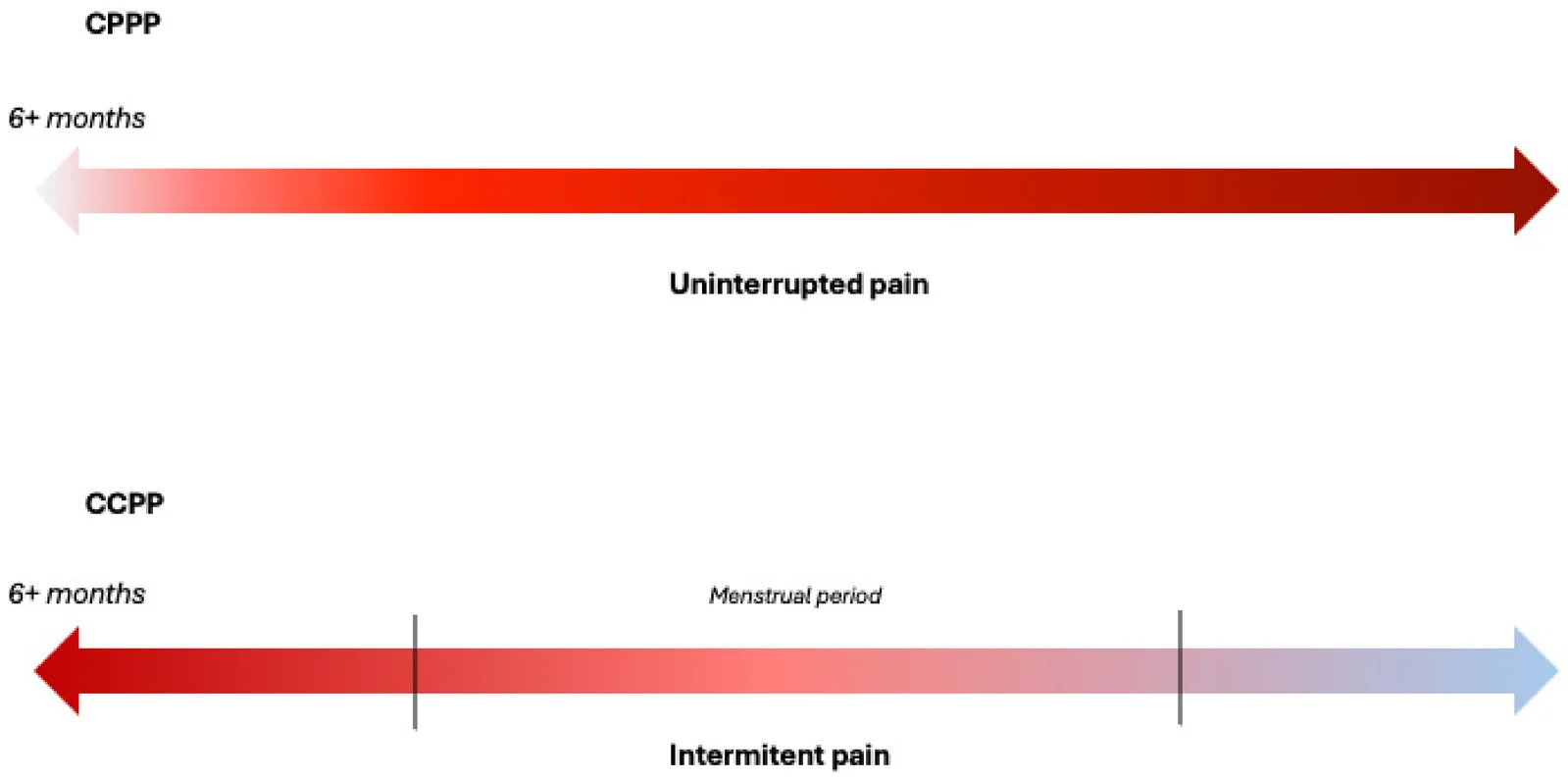
Chronic Permanent Pelvic Pain and Chronic Cyclic Pelvic Pain classification. CPPP is classified as uninterrupted pelvic pain for 6 months or more, whereas CCPP is classified as intermittent pelvic pain that presents before menstrual period and decreases its intensity after the period for a duration of 6 months or more. Abbreviations: +, more; CPPP, Chronic Permanent Pelvic Pain; CCPP, Chronic Cyclic Pelvic Pain.

An exploratory, observational, descriptive, cross-sectional, comparative with repeated sampling study was carried out at the Instituto Nacional de Perinatología (INPer) in Mexico City during the years 2023 to 2025. The study was approved by the Research, Ethics, and Biosafety Committees of the Institute (Reg. 2023-1-13), by the tenets of the Declaration of Helsinki for research involving human subjects. Fifty-two patients were included and divided into two groups based on the type of pain: the Chronic Permanent Pelvic Pain (CPPP) group (*n* = 33), and the Chronic Cyclic Pelvic Pain (CCPP) group (*n* = 19). Sample size was determined by using G-power to detect a moderate effect size (Cohen’s d = 0.54) with a power of 61% for CPPP and 70% for CCPP with an α = 0.05. Women who the Gynecologic and Obstetric Department followed up due to an endometriosis diagnosis were referred to the Neuroscience Department for mental care support due to anxious/depressive behavior. The patients’ diagnoses were based on imaging mapping (ultrasound and magnetic resonance imaging) and clinical criteria (symptoms) [44]. Subsequently, the ENZIAN stratification system was used [45]. The time to reach a diagnosis was 4 months. All participating patients presented with infertility secondary to the disease, without prior surgery for the treatment and control of endometriosis. All subjects were treated with hormonal medication. Those who needed this attention for the management of anxiety/depression symptoms received psychoeducation and emotional support. Patients between 18 and 60 years old were invited to participate in the study by signing a written informed consent. Afterward, patients applied a semi-structured clinical history, which included sociodemographic and clinical data. The sample was clustered according to their type of CPP. Clustering was performed by a clinical questionnaire, which includes the criteria of 6 months or more of uninterrupted pelvic pain to be classified as CPPP, while 6 months or more of intermittent pain that occurs before the menstrual period, increases during the period, and decreases a few days after the period ends can be considered as CCPP (Figure 1) and is illustrated in Figure 2 [46].

In the image: the CPPP section represents pain as follows: a lighter color indicates less pain; the darker the arrow, the greater the perceived pain. The CCPP section represents intermittent pain as follows: prior to menstruation, a bright red color indicates an increased perception of pain that persists during the first few days of menstruation and decreases over time until it disappears once menstruation has ended, which is represented by a change to blue.

### 2.2. Saliva Sample Collection

Participants were instructed to abstain from alcohol and tobacco consumption for at least 24 h prior to sample collection. Additionally, during the hour preceding sampling, they were asked to refrain from eating, drinking, brushing their teeth, using lip balm or salve, or engaging in any activity that could compromise oral integrity. The samples were taken at 9:00 am in a sample collection room; all saliva samples were taken during the first day of the participants’ menstrual period. The participants had a history of treatment with analgesics; however, they were asked not to take them 8 h before the sample was taken, and none of the participants were under psychopharmacological treatment. The dental service ruled out any issues related to oral health management or dental infections; however, the existence of a lesion prior to the examination could not be controlled. A brief assessment was also conducted to rule out the presence of local infections at the time of collection. Unstimulated whole saliva (4 mL) was collected into sterile 15 mL centrifuge tubes at three time points: baseline, post-stressor, and recovery. For the stress stage, the Trier Social Stress Test (TSST) was used, which is designed to induce acute stress and consists of a simulated job interview and a mental arithmetic calculation in front of a group of interviewers to measure physiological and psychological responses with 15 min intervals between each collection [47]. Samples were kept at 4 °C immediately after collection and transported to the laboratory, where they were centrifuged at 1000× *g* for 10 min. The supernatant was carefully aliquoted into 2 mL microcentrifuge tubes and stored at −80 °C until analysis.

### 2.3. Quantification of IL-1β, IL-6, and TNF-α

Cytokine concentrations (IL-1β, IL-6, and TNF-α) were determined using DuoSet^®^ ELISA kits (R&D Systems, Minneapolis, MN, USA; IL-1β Cat# DY201, Lot P381233; IL-6 Cat# DY206, Lot P411622; TNF-α Cat# DY210, Lot P406138). Standard curves were generated using a four-parameter logistic fit (R^2^ > 0.99). The limits of detection (LODs) were 3.91 pg/mL for IL-1β, 9.38 pg/mL for IL-6, and 15.6 pg/mL for TNF-α, while the limits of quantification (LOQs), established according to ICH Q2 (R1) criteria, were 12.5, 31.3, and 52.0 pg/mL, respectively. All measured concentrations exceeded these thresholds, confirming that the assays were performed within the validated quantitative range. Previously published salivary cytokine concentrations were consulted solely as background context and were not used as an external control group or for inferential statistical comparisons. Therefore, the variability observed among participants reflects true biological differences rather than analytical noise.

Saliva was chosen as the biological matrix because it allows non-invasive and stress-free sampling, which is especially important in studies involving women with chronic pelvic pain. Previous works [48,49,50] support the use of saliva as a reliable and reproducible fluid for cytokine quantification, provided that pre-analytical conditions are strictly controlled. All samples were processed within 20 min of collection, centrifuged, and stored at −80 °C with a single freeze–thaw cycle, ensuring optimal preservation of cytokine integrity. All measured concentrations were above the analytical limits of quantification (IL-1β: 12.5 pg/mL; IL-6: 31.3 pg/mL; TNF-α: 52.0 pg/mL), confirming that the assays were technically sound and that the observed differences reflect intrinsic biological variability rather than analytical noise. It is important to note that salivary cytokine levels can be influenced by individual, hormonal, and environmental factors, and this inherent variability represents a limitation of the study. Even so, under the standardized conditions applied in our research, saliva provides a robust and ethically acceptable alternative for monitoring inflammatory activity in women with endometriosis and chronic pelvic pain.

### 2.4. Mental Status Assessment Pain Behavior

To determine the types of anxiety, patients were measured by the State-Trait Anxiety Inventory (STAI), a 40-item questionnaire standardized for Mexican women [51]. The cut-off was: 47 points for trait anxiety and 43 points for state anxiety [52]. For depression symptoms, a 30-item Goldberg General Health Questionnaire (GHQ-30) [53,54] was translated, adapted, and validated for this population. A score of <7 points was considered the absence of depression, while a score >8 points was considered compatible with the presence of depression [55,56]. To classify the depression symptoms, a Beck Depression Inventory (BDI-II) was applied to our population and used to assess depressive traits, consisting of 21 items with cut-off points as follows: 0–9 absent, 10–16 mild depression, 17–29 moderate depression, and greater than 30 severe depression [57]. Also a Hospital Anxiety and Depression Scale (HADS), a 14-item questionnaire validated for our population, was used to evaluate their in-hospital emotional distress (anxiety and depression symptoms), scored on a 4-point Likert frequency scale (0–3), with a total score ranging from 0 to 21 in each subscale, where a higher score indicates greater severity of symptoms [58], scores of 0–7 showed absence of symptoms, 8–10 moderate symptoms, and 11–21 severe symptoms [59]. To assess cognitive impairment in these women, a 30-item Mini-Mental State Examination (MMSE) was administered, with higher scores indicating less cognitive impairment [60,61,62]. Scores between 27 and 30 indicate no cognitive impairment. Scores between 24 and 26 are suspicious for cognitive impairment. Scores between 23 and 12 points are indicative of mild-to-moderate cognitive impairment. Patients with scores of 11–9 points have severe cognitive impairment. The Wong–Baker Pain Rating Scale (FACES) [63] was used for the assessment of pain intensity. Six-drawn faces with scores ranging from 0 to 10 were included; the identification according to the sensation of pain is graphically represented with the faces and quantitatively with the values represented by each of them, as follows 0—“No Hurt”, 2—“Hurts a Little Bit”, 4—“Hurts a Little More”, 6—“Hurts Even More”, 8—“Hurts Whole Lot” and 10—“Hurts Worst” [64]. From the evaluation of the study, those patients who were diagnosed with anxiety and/or depression or emotional distress, and/or pain had access to psychological care through the neuroscience department.

### 2.5. Statistical Analysis

Normality of continuous variables was assessed using the Shapiro–Wilk test. To formally test moderation by pain type, ordinary least squares (OLS) regression models were estimated as: outcome ~ TNF-α + group + TNF-α × group, with CPPP as the reference. Fisher’s z tests were used to compare Spearman coefficients across subgroups. These analyses were conducted post hoc and are considered exploratory. Variables showing normal distribution (GHQ-30, HADS total score and STAI-State anxiety in both groups; all *p* > 0.05) were compared between groups using Welch’s independent-samples t-test. Non-normally distributed variables (BDI, STAI-Trait, FACES, and all salivary cytokines) were compared using the Mann–Whitney U test. Categorical variables (presence/absence of depressive symptoms, emotional distress, and anxiety) were analyzed using chi-square tests of independence. Associations between psychological variables and salivary cytokine levels were evaluated using Spearman’s rank correlation coefficient. To control for multiple comparisons, the false discovery rate (FDR) was controlled using the Benjamini–Hochberg procedure across each family of tests. All analyses were performed in Python 3 (Python Software Foundation, Beaverton, OR, USA) and are publicly available as a reproducible Jupyter notebook. Statistical significance was set at *p* < 0.05.

## 3. Results

### 3.1. Study Population

Participants. A total of 52 women with confirmed endometriosis were included: 33 with Chronic Permanent Pelvic Pain (CPPP) and 19 with Chronic Cyclic Pelvic Pain (CCPP). No statistically significant difference between groups in age was observed (CPPP: 36.4 ± 6.2 years; CCPP: 35.3 ± 7.8 years). Table 1 summarizes the clinical and psychological characteristics of the CPPP and CCPP groups.

### 3.2. Mental Status Assessment

Cognitive screening. All participants showed intact cognitive function on the MMSE, with no significant difference between groups (CPPP: Mdn = 30 [IQR: 29–30]; CCPP: Mdn = 30 [IQR: 29–30]; U = 264, *p* = 0.347).

Psychological variables. No statistically significant differences between groups were found in any psychological measure after FDR correction. HADS total scores were 10.2 ± 7.2 (CPPP) vs. 11.1 ± 7.4 (CCPP; t = −0.41, *p* = 0.682). BDI scores were Mdn = 14 [IQR: 8–25] vs. Mdn = 17 [IQR: 10–22] (U = 324, *p* = 0.842). STAI-State anxiety was 41.2 ± 5.5 vs. 43.9 ± 5.4 (t = −1.72, *p* = 0.086), STAI-Trait was Mdn = 46 [IQR: 41–50] vs. Mdn = 48 [IQR: 43–51] (U = 287, *p* = 0.615), GHQ-30 was 10.9 ± 8.6 vs. 10.0 ± 8.9 (t = 0.35, *p* = 0.730), and pain intensity on the FACES scores was Mdn = 6 [IQR: 4–8] vs. Mdn = 4 [IQR: 4–8] (U = 333.5, *p* = 0.705).

These results indicate that CPPP and CCPP groups did not differ significantly in emotional state, pain perception, or general psychological distress. These patterns are consistent with the descriptive statistics presented in Table 1.

Categorical psychiatric indicators. The proportion of women meeting the BDI threshold for clinically significant depressive symptoms (score ≥ 10) was similar between groups: 69.7% (23/33) in CPPP vs. 73.7% (14/19) in CCPP (χ^2^ = 0.093, df = 1, *p* = 0.760). No significant between-group differences were observed in the prevalence of emotional distress on the HADS (48.5% vs. 73.7%; χ^2^ = 3.14, *p* = 0.077), state anxiety (54.5% vs. 52.6%; χ^2^ = 0.018, *p* = 0.894), or trait anxiety (69.7% vs. 73.7%; χ^2^ = 0.093, *p* = 0.760). Table 2 summarizes the prevalence of clinically significant depressive symptoms and other psychological indicators.

### 3.3. Evaluation of Inflammatory Biomarkers

**Figure 3:**
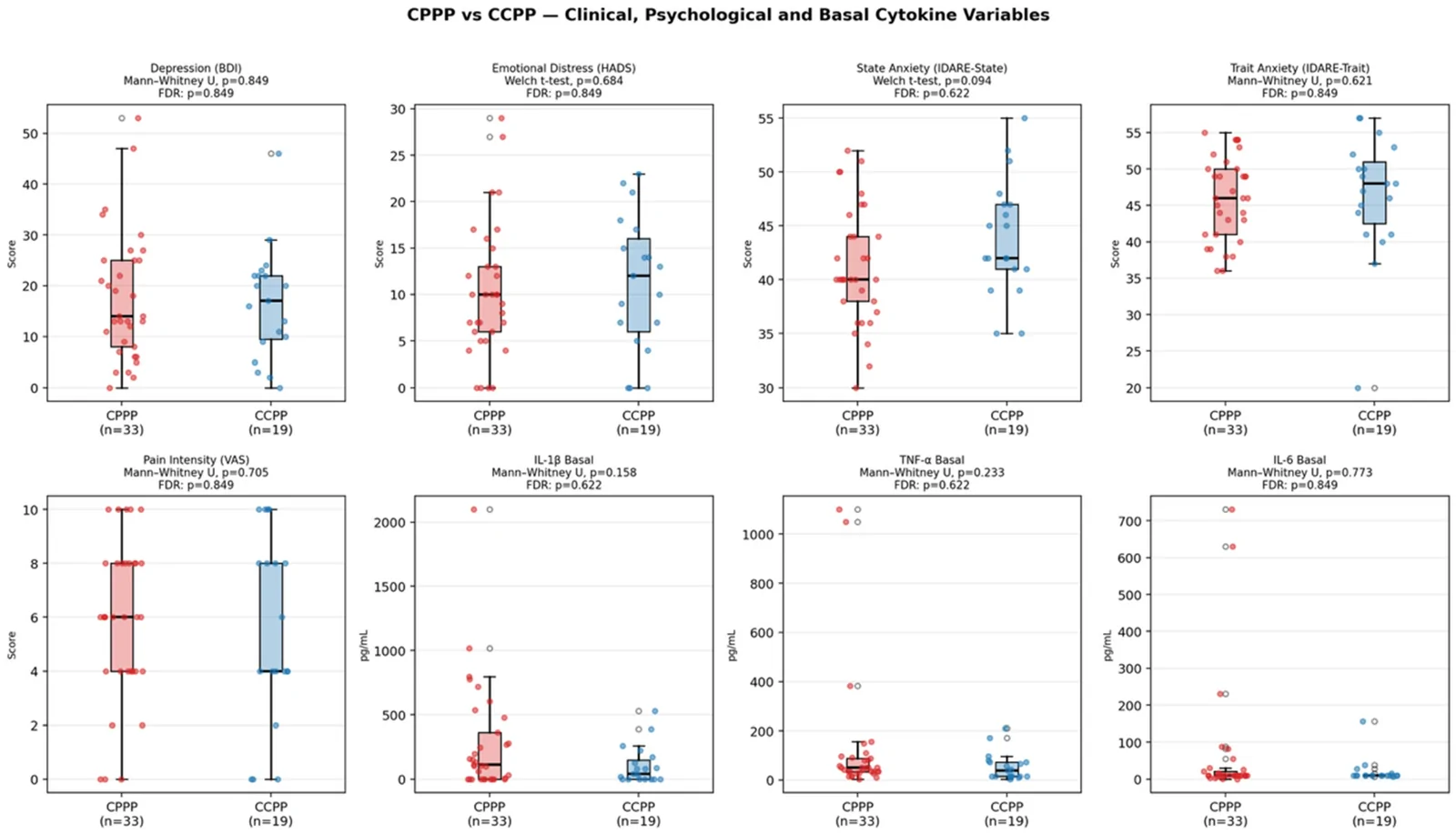
Comparison of clinical, psychological, and basic inflammatory biomarkers in women with chronic permanent pelvic pain (CPPP) and chronic cyclic pelvic pain (CCPP). The boxplots display scores from the Beck Depression Inventory (BDI), State-Trait Anxiety Inventory (STAI-State and STAI-Trait), Hospital Anxiety and Depression Scale (HADS), FACES, and levels of salivary cytokines (IL-1β, IL-6, and TNF-α) in women with endometriosis, grouped by pain pattern. After correcting for false discovery rate, there were no significant differences between the groups. Abbreviations: BDI, Beck Depression Inventory; CPPP, Chronic Permanent Pelvic Pain; CCPP, Chronic Cyclic Pelvic Pain; HADS, Hospital Anxiety and Depression Scale; IL, interleukin; STAI, State-Trait Anxiety Inventory; TNF-α, tumor necrosis factor alpha.

Salivary levels of cytokines IL-1β, IL-6, and TNF-α levels at baseline, under stress, and during recovery did not differ significantly between CPPP and CCPP groups (all Mann–Whitney U tests, all *p* > 0.05 after FDR correction. Although CPPP women showed numerically higher IL-1β at baseline (Mdn = 111.9 [IQR: 0.5–365.2] pg/mL) compared to CCPP (Mdn = 41.1 [IQR: 0.5–148.4] pg/mL; U = 387, *p* = 0.158), this difference did not reach statistical significance. IL-6 levels were similarly distributed across conditions and groups. TNF-α concentrations were also comparable between groups at all time points (Basal: U = 250, *p* = 0.231; Stress: U = 272, *p* = 0.436; Recovery: U = 260, *p* = 0.309). Because no matched healthy control group was included, these findings should be interpreted as within cohort comparisons between CPPP and CCPP groups, without inferring whether cytokine concentrations were increased or decreased relative to healthy women. Figure 3 illustrates the distribution of clinical, psychological and basal cytokine variables for both groups.

### 3.4. Clinical Correlations

**Figure 4:**
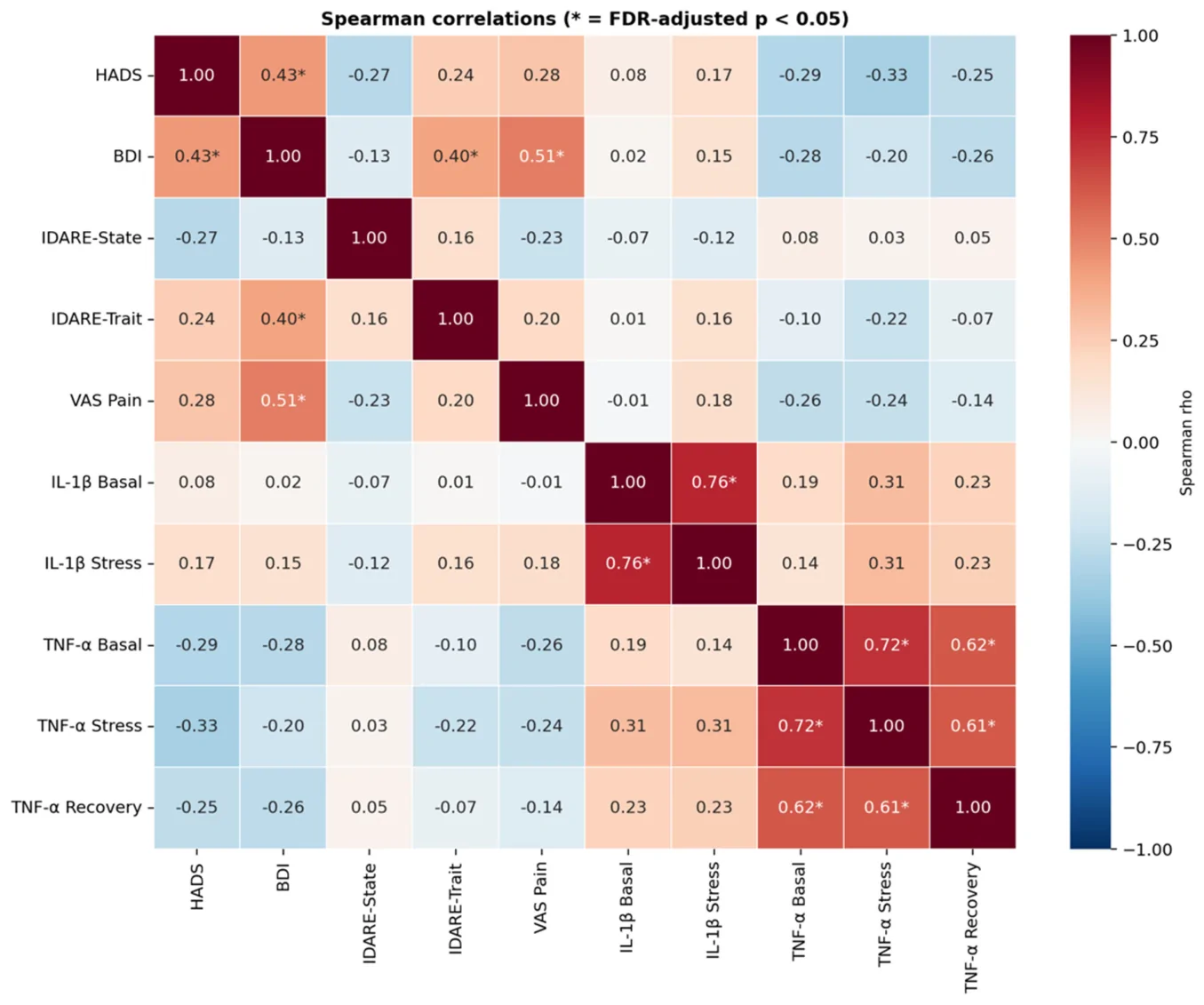
Correlation matrix between emotional state variables and salivary inflammatory biomarkers in women with endometriosis. A heatmap displays Spearman correlation coefficients between psychological variables, pain perception, and salivary cytokine concentrations at baseline, stress, and recovery. At the uncorrected level, exploratory inverse associations were observed between TNF-α concentrations and emotional distress indicators (HADS, BDI, GHQ-30); however, none survived FDR correction (all p_FDR < 0.10). After FDR adjustment, only within-domain correlations remained significant (psychological variables with each other, and cytokine timepoints with each other). Asterisks (*) denote FDR adjusted *p* < 0.05. Abbreviations: BDI, Beck Depression Inventory; GHQ-30, Goldberg General Health Questionnaire score; HADS, Hospital Anxiety and Depression Scale; IL, interleukin; TNF-α, tumor necrosis factor alpha.

Correlations between emotional state and salivary cytokines are presented. Spearman’s rank correlation analyses revealed significant negative associations between TNF-α levels and measures of emotional distress across the sample (*n* = 52). At the uncorrected significance level (*p* < 0.05), TNF-α at baseline was inversely associated with HADS total score (ρ = −0.292, *p* = 0.031) and BDI score (ρ = −0.278, *p* = 0.041). TNF-α under stress conditions showed negative associations with HADS (ρ = −0.329, *p* = 0.014) and GHQ-30 (ρ = −0.271, *p* = 0.046), and TNF-α during recovery was inversely associated with GHQ-30 (ρ = −0.363, *p* = 0.006). However, none of these associations survived FDR correction (all p_FDR < 0.10) and should therefore be considered exploratory findings. The correlations that remained statistically significant after FDR adjustment were confined to within-domain associations (psychological variables with each other, and cytokine timepoints with each other), as detailed in Figure 4.

An exploratory stratified analysis was conducted to examine whether pain chronification type moderated the relationship between salivary TNF-α and psychological burden. When Spearman correlation were computed separately within each subgroup (*n* = 19), TNF-α levels—measured at baseline, stress, and recovery—showed consistent negative associations with BDI (TNF-α Basal: ρ = −0.563, *p* = 0.005; TNF-α Stress: ρ = −0.573, *p* = 0.004), GHQ-30 (TNF-α Basal: ρ = −0.484, *p* = 0.023; TNF-α Stress: ρ = −0.618, *p* = 0.001; TNF-α Recovery: ρ = −0.435, *p* = 0.046), HADS (ρ = −0.467, *p* = 0.030), and pain intensity (FACES × TNF-α Basal: ρ = −0.517, *p* = 0.013). In contrast, no significant associations between TNF-α and psychological variables were observed in the CPPP subgroup (*n* = 33). To formally test whether pain chronification type moderated the TNF-α–psychological burden association, OLS regression models were specified as: psychological variable ~ TNF-α + group + TNF-α × group (CPPP = 0, CCPP = 1). Significant negative interaction terms were observed for BDI across all TNF-α conditions (Basal: β = −0.126, t = −2.57, *p* = 0.013; Stress: β = −0.081, t = −2.52, *p* = 0.015; Recovery: β = −0.068, t = −2.12, *p* = 0.039), for GHQ-30 across all conditions (Basal: β = −0.093, t = −2.61, *p* = 0.012; Stress: β = −0.058, t = −2.49, *p* = 0.016; Recovery: β = −0.050, t = −2.15, *p* = 0.036), and for HADS at baseline (β = −0.075, t = −2.52, *p* = 0.015). Complementary Fisher’s z tests confirmed significantly different Spearman coefficients between groups for BDI × TNF-α Stress (z = 2.24, *p* = 0.025), GHQ-30 × TNF-α Basal (z = 2.10, *p* = 0.036), and GHQ-30 × TNF-α Stress (z = 2.28, *p* = 0.022). These results provide formal statistical support for the hypothesis that pain chronification type moderates the TNF-α–psychological burden relationship. Because these analyses were conducted post hoc and were exploratory in nature, unadjusted *p* values are reported and should be interpreted cautiously. Given the sample size of the CCPP subgroup (*n* = 19), replication in larger independent samples will be necessary before confirmatory conclusions can be drawn.

## 4. Discussion

Pain caused by endometriosis is a recurrent main problem; in some cases, women with endometriosis in a reproductive age are affected in their reproductive rights [65], resulting in a perception of a catastrophic scenario such as infertility, disabling pelvic pain and in most of the cases, emotional dysregulation, affecting a favorable prognosis, which poses a medical, psychological, social and family challenge for women [66].

This condition creates uncertainty and modifies effective communication with those around patients with endometriosis. They often adopt avoidant behaviors due to emotional, family, and social stigma, which leads to the development of emotional distress symptoms, such as anxiety and depression. This condition can be explained by the presence of high levels of pain [67,68]. The findings of this study align with the existing literature, demonstrating that avoidant behaviors and impaired communication are associated with pain-related disability. Significant features of psychological distress observed in this cohort further support these conclusions. In our cohort, the high prevalence of clinically significant depressive, anxious and distress symptoms shown in Table 2 reflects the psychological burden associated with endometriosis.

In women who presented with characteristics of psychological distress and difficulty in adhering to medical treatment, it was found that adequate information management, as well as social and family support, functioned as protective factors [69]. This is consistent with our study, despite having adequate information about diagnosis and treatment, a high level of education, and social and family support. However, this does not always mean an improvement in the physical and mental health of patients.

In addition, the development of features of psychological distress, as well as symptoms of anxiety and depression, can be influenced by other factors such as dysfunction in the couple’s relationship, ineffective assertive communication, changes in the perception of pain, difficulties in personal and couple sexual life, infertility, and a lack of emotional, social and economic support [70]. These phenomena were present in a large proportion of the women, which may explain the denial of the problem, resulting in physical and/or psychological symptoms that, in some cases, generate social and familial stigmatization, leading to the presence of violence in the couple’s relationship, as our results suggest.

To prevent and treat psychological distress or other mental disorders during the disease, effective coping is necessary. This approach should be directed at the source of the problems, encouraging patients to use their intrapersonal resources to resolve such conditions by adapting appropriately to their social, family, and partner environment, all of this together with specialized medical and psychological support [71]. Although effective coping consists of psychotherapy and/or psychopharmacological treatment, cognitive behavioral therapy, especially acceptance and commitment therapy, is one of the essential tools in pain management. Psychopharmacological treatment is used only in cases where there is no improvement in the symptomatology of psychological distress [72]. We recommend implementing these strategies permanently to ensure effective management of symptoms that may lead to limitation, as this is often not the case in our study. The exacerbation of anxiety and depression symptoms in patients living with endometriosis may be due to the perception of their disease and the process of coping with it [73,74], even when psychotherapy was applied after diagnosis, as this fact is a limitation of our study; this phenomena may be explained by the patient’s perception of disease, as patients developed unrealistic expectations regarding treatment outcomes, their medical team and the outcome of the disease.

Regarding the quantification of salivary biomarkers of inflammation, our results showed that there is only a significant difference in the concentration of IL-1β in its basal state. This can be explained by the presence of IL-1β in chronic inflammatory processes. IL-1β also functions as a precursor of IL-6, which may be related to mood alterations. This should be taken as a possible trend rather than a statistically significant assertion. An important limitation of this study is the absence of a matched healthy control group, which limits the interpretation of salivary cytokine concentrations relative to physiological reference values. Previously published salivary cytokine concentrations were therefore used only as contextual background and not as a basis for direct quantitative or statistical comparison. Differences in saliva collection procedures, sample processing, ELISA platforms, analytical sensitivities, and participant characteristics preclude direct comparisons across studies. Accordingly, our findings should be interpreted as an exploratory study within cohort observations comparing women with chronic permanent pelvic pain and chronic cyclic pelvic pain, rather than as evidence of increased or decreased salivary cytokine concentrations relative to healthy women. Our present findings establish the ground for future studies, as including age- and reproductive status-matched healthy controls, processed under identical pre-analytical and analytical conditions, is required to establish disease-specific reference intervals. Further, the cytokine concentrations observed in our participants confirm measurable salivary inflammation activity within this cohort; however, in the absence of matched healthy controls, they cannot be classified as elevated or reduced relative to healthy women. The reliability of inflammatory biomarkers in heterogeneous endometriosis populations deserves careful consideration. As demonstrated by Zyguła et al., the leptin/BMI ratio in plasma and peritoneal fluid did not differ significantly between women with endometriosis and controls, despite some subgroup signals related to primary infertility. This finding underscores that biologically plausible markers do not always perform as reliable diagnostic biomarkers in heterogeneous patient populations, a caveat equally applicable to our salivary cytokine findings, where group-level differences did not reach significance after FDR correction. Individual variability in inflammatory responses, influenced by phenotypic heterogeneity, hormonal status, and unmeasured confounders, likely contributes to the wide overlap observed in cytokine distributions across groups [75].

Consistent with recent research on inflammatory biomarkers in endometriosis, our findings support the concept that immune dysregulation plays a role in the disease while also highlighting the challenges associated with biomarker interpretation. Previous studies evaluating proteasome, immunoproteasome, and poly (ADP-ribose) polymerase (PARP) in plasma and peritoneal fluid have reported measurable alterations in immune and stress response pathways in women with endometriosis, both locally within the peritoneal cavity and systemically [76]. Although these observations strengthen the immunological basis of endometriosis, they also emphasize the need for careful interpretation and validation of potential biomarkers in well designed, controlled populations.

Within this framework, the salivary biomarkers evaluated in our study (IL-1β, IL-6, and TNF-α) provide a complementary and completely non-invasive approach for assessing low-grade inflammatory activity. However, unlike biomarkers measured in plasma or peritoneal fluid, salivary cytokine concentrations may be influenced by factors such as oral health status, lifestyle habits, and sample collection or processing conditions, limiting direct quantitative comparisons across biological compartments [48]. Therefore, we do not suggest that salivary cytokines should be used as independent diagnostic markers or as direct substitutes for established measures such as PARP. Rather, our results should be viewed as preliminary evidence indicating that chronic pelvic pain associated with endometriosis may be accompanied by subtle inflammatory signals detectable in saliva. Further studies involving larger, well controlled cohorts, including healthy controls, are needed to confirm these findings and clarify their potential clinical relevance.

We can deduce that the correlations between symptoms of anxiety, emotional discomfort, and symptoms of depression are highly interrelated; likewise, a nonsignificant trend was observed wherein TNF-α concentrations at baseline and during recovery were lower in participants with more pronounced anxiety symptoms, though this pattern should not be interpreted as a confirmed association given the absence of FDR-corrected significance and the limited sample size. With regard to the remaining concentrations of inflammatory biomarkers in saliva, they showed no significant difference; this could be due to various situations such as poor oral health care, regional oral infection, dietary habits high in sugar and fat, consumption of stimulating drinks, chronic and inflammatory diseases, and obesity, among others [77], which we consider to be complex variables to control, despite the clear instructions given to patients. Moreover, various studies show that although it is possible to quantify inflammatory biomarkers in saliva, there are limitations such as standardization of saliva collection and analysis, low accuracy, and poor reference ranges [55]. In contrast, we consider saliva samples to avoid more pain and stress in our patients, trying to prevent alterations in biomarker levels.

A plausible biological explanation for the inverse association between TNF-α and psychological burden observed exclusively in the CCPP subgroup may involve the hypothalamic–pituitary–adrenal (HPA) axis. In the context of cyclic pain, the inflammatory response retains a degree of temporal variability that allows the HPA axis to remain reactive: elevated psychological distress activates the HPA axis, increasing cortisol secretion, which in turn suppresses pro-inflammatory cytokine production, including TNF-α [78,79]. This neuroendocrine negative feedback loop may explain the inverse correlation pattern observed exclusively in CCPP women. In contrast, patients with continuous pelvic pain are exposed to sustained nociceptive and psychological stressors that may lead to HPA axis dysregulation, a phenomenon documented in both chronic pain conditions and endometriosis specifically characterized by blunted cortisol reactivity and disruption of the typical cortisol–cytokine regulatory relationship [80,81]. Under these conditions, TNF-α levels may become decoupled from psychological state, accounting for the absence of the inverse pattern in the CPPP subgroup. This interpretation is necessarily speculative since in our study we did not directly measure the function of the HPA axis, which justifies a direct verification by simultaneous measurement of cortisol and cytokine trajectories in future studies.

These results expand the perspective on integrative treatment for women with endometriosis by highlighting the importance of equipping this population with personal tools for effective coping, pain perception management, and the strengthening of social, familial, and partner support. Psychotherapeutic interventions are identified as appropriate solutions. The identification and modulation of inflammatory biomarkers facilitate the assessment of psychotherapeutic effectiveness, serving as a protective factor as supported by the literature. It is recommended to develop criteria for identifying mental health needs and to establish standardized reference ranges for the quantification of inflammatory biomarkers (Table 3).

One of the main strengths of this study lies in its integrative approach, combining psychometric assessment with the quantification of salivary proinflammatory cytokines. This design enabled a multidimensional analysis of CPP in women with endometriosis, linking subjective experiences of pain and emotional distress with objective biological markers. Another notable strength is the inclusion of a naturalistic stressor and sequential sampling, which provided insights into the dynamic behavior of inflammatory markers across different physiological states. However, this study also presents some limitations. The sample size, although sufficient for exploratory analysis, limits the generalizability of the findings and the statistical power for detecting significant differences in biomarker levels and subtle associations. Additionally, the cross-sectional nature of the cytokine assessments prevents conclusions about causal relationships between inflammation and emotional symptoms. The absence of a healthy control group also restricts the interpretation of cytokine levels within a broader physiological context. Finally, potential confounding factors such as hormonal treatments or comorbid conditions were not fully controlled, which may have influenced the inflammatory profiles observed. Furthermore, the distribution of disease phenotypes including deeply infiltrating endometriosis (DIE), endometrioma, and superficial endometriosis as well as ENZIAN locations, rASRM/ENZIAN stage, severity of dysmenorrhea, dyspareunia, dyschezia, and dysuria, duration of symptoms, and the specific hormonal treatments used, were not systematically reported. This represents a significant limitation, as phenotypic heterogeneity may have influenced both psychological and inflammatory outcomes, and should be addressed in future studies with complete clinical characterization. Several potential confounders were not included in the statistical models, including BMI, smoking status, dietary habits, specific hormonal treatment type, NSAID and analgesic use beyond the 8 h withdrawal period, oral health status, and systemic infections. The 8 h washout for analgesics and anti-inflammatory drugs is particularly limiting, as many NSAIDs have pharmacological half-lives exceeding this window and could plausibly influence salivary cytokine profiles. Future studies should incorporate these variables as covariates in multivariate regression models. Pain intensity was assessed using FACES, which, while widely used in clinical settings for its accessibility, is considered a suboptimal instrument for adult women with endometriosis. More comprehensive tools such as the Numerical Rating Scale (NRS), Brief Pain Inventory (BPI), Endometriosis Health Profile30 (EHP30), and the Central Sensitization Inventory, with separate evaluation of dysmenorrhea, dyspareunia, dyschezia, dysuria, and non-menstrual pelvic pain, would provide a more nuanced characterization of the pain experience in this population. This represents a methodological limitation of the current study that will be addressed in future research.

Despite these limitations, the study offers valuable evidence supporting the biopsychosocial model of endometriosis and highlights the need for interdisciplinary approaches in both research and clinical care.

## 5. Conclusions

This study adds to the evidence highlighting the complex interplay between psychological symptoms and immune responses in women with endometriosis. No statistically significant between group differences in depressive symptoms or salivary inflammatory markers were found after FDR correction; however, given the limited statistical power (61–70%), these results should not be interpreted as evidence of equivalence between groups. However, a tendency was shown. Furthermore, the observed inverse trend between TNF-α levels and markers of emotional dysregulation underscores the relevance of neuroimmune interactions in the affective dimensions of chronic pain and warrants further investigation in larger samples. These results emphasize the necessity of moving beyond symptom-based treatment toward integrative care models that address both biological and psychological aspects of endometriosis. Future research should investigate whether targeted psychological or pharmacological interventions can modulate inflammatory responses and improve clinical outcomes in this population. The perception of pain may increase the risk of developing symptoms of emotional dysregulation, anxiety, and depression. Exploratory analyses suggested patterns between salivary inflammation and psychological burden; however, these findings should be interpreted cautiously because of the small subgroup size, multiple comparisons, and lack of a matched healthy control group.

## Figures and Tables

**Table 1:** Clinical and psychological characteristics of women with endometriosis according to pain pattern. Data are presented as mean ± standard deviation (SD) or median [interquartile range (IQR)], according to data distribution. Group comparisons were performed using Welch’s independent-samples *t*-test or Mann–Whitney U test, as appropriate. Statistical significance was set at *p* < 0.05. Abbreviations: CPPP, Chronic Permanent Pelvic Pain; CCPP, Chronic Cyclic Pelvic Pain; MMSE, Mini-Mental State Examination; HADS, Hospital Anxiety and Depression Scale; BDI-II, Beck Depression Inventory-II; STAI-State, State Anxiety Inventory; STAI-Trait, Trait Anxiety Inventory; GHQ-30, Goldberg General Health Questionnaire-30; FACES; IQR, Interquartile Range; SD, Standard Deviation.

Variable	CPPP (*n* = 33)	CCPP (*n* = 19)	Test	Statistic
BDI-II	14.00 [8.00–25.00]	17.00 [9.50–22.00]	Mann–Whitney U	324.0
HADS total	10.24 ± 7.22	11.11 ± 7.36	Welch *t*-test	−0.41
STAI-State	41.21 ± 5.49	43.89 ± 5.37	Welch *t*-test	−1.72
STAI-Trait	46.00 [41.00–50.00]	48.00 [42.50–51.00]	Mann–Whitney U	287.0
GHQ-30	10.91 ± 8.62	10.00 ± 8.89	Welch *t*-test	0.35
FACES	6.00 [4.00–8.00]	4.00 [4.00–8.00]	Mann–Whitney U	333.5

**Table 2:** Prevalence of clinically significant psychological symptoms in CPPP and CCPP groups. Data is presented as number (percentage). Group comparisons were performed using chi-square (χ^2^) tests of independence. Statistical significance was set at *p* < 0.05. Abbreviations: CPPP, Chronic Permanent Pelvic Pain; CCPP, Chronic Cyclic Pelvic Pain; BDI-II: Beck Depression Inventory-II; HADS, Hospital Anxiety and Depression Scale; χ^2^, Chi-square test.

Variable	CPPP *n* (%)	CCPP *n* (%)	χ^2^	*p*
Depressive symptoms (BDI ≥ 10)	23 (69.7)	14 (73.7)	0.093	0.760
Emotional distress (HADS)	16 (48.5)	14 (73.7)	3.14	0.077
State anxiety	18 (54.5)	10 (52.6)	0.018	0.894
Trait anxiety	23 (69.7)	14 (73.7)	0.093	0.760

**Table 3:** Significant Spearman correlations between clinical and psychological variables and inflammatory biomarkers after FDR correction. We used Spearman rank correlation analyses to examine the links between psychological variables, pain perception, and salivary cytokine levels in women with endometriosis. *p*-values were adjusted with the Benjamini–Hochberg false discovery rate (FDR) correction. Only correlations that remained statistically significant after FDR adjustment are included. Abbreviations: BDI-II, Beck Depression Inventory-II; FACES; FDR, False Discovery Rate; HADS, Hospital Anxiety and Depression Scale; IL-1β, Interleukin-1 beta; TNF-α, Tumor Necrosis Factor alpha.

Variable 1	Variable 2	Spearman’s ρ	Raw *p*-Value	FDR-Adjusted *p*-Value
HADS	BDI-II	0.433	0.001	0.010
BDI-II	FACES	0.510	<0.001	0.002
IL-1β Basal	IL-1β Stress	0.760	<0.001	<0.001
TNF-α Basal	TNF-α Stress	0.720	<0.001	<0.001
TNF-α Basal	TNF-α Recovery	0.620	<0.001	<0.001
TNF-α Stress	TNF-α Recovery	0.610	<0.001	<0.001

## Data Availability

The data and psychometric instruments are available. To obtain access to the raw data you must contact María del Pilar Meza-Rodríguez by email: mezapilar@yahoo.com; data is available upon reasonable request.

## Abbreviations

CCPP	Chronic Cyclic Pelvic Pain
CPPP	Chronic Permanent Pelvic Pain
IL-1β	Interleukin-1β
IL-6	Interleukin-6
TNF-α	Tumor Necrosis Factor Alpha
STAI	State/Trait Anxiety Inventory
BDI-II	Beck Depression Inventory
GHQ-30	Goldberg General Health Questionnaire
HADS	Hospital Anxiety and Depression Scale
MMSE	Mini-Mental State Examination
FACES	Wong–Baker Pain Rating Scale
FDR	False Discovery Rate
HPA	Hypothalamic–Pituitary–Adrenal
INPer	Instituto Nacional de Perinatología
CPP	Chronic Pelvic Pain
DHEA-S	Sulfated Dehydroepiandrosterone
CBTs	Cognitive Behavioral Therapies
ACT	Acceptance and Commitment Therapy
ELISA	Enzyme-linked Immunosorbent Assay
CVs	Coefficients of Variations
OLS	Ordinary Least Squares
STAI-S	State/Trait Anxiety Inventory—State
STAI-T	State/Trait Anxiety Inventory—Trait
